# Elevated cytokines in osteoporotic blood correlate with immune microenvironment shifts and dysregulated circRNA profiles

**DOI:** 10.3389/fimmu.2026.1914272

**Published:** 2026-09-09

**Authors:** Ying Wang, Yingqing Chen, Ali Deng, Shuang Wu, Ni Zhan, Yingjun Deng, Yiti Huang, Mengxin Xiong, Jun Xu

**Affiliations:** 1 Hubei University of Chinese Medicine, Wuhan, China; 2 Hubei Provincial Hospital of Traditional Chinese Medicine, Wuhan, China; 3 Affiliated Hospital of Hubei University of Chinese Medicine, Wuhan, China; 4 Hubei Key Laboratory of Theory and Application Research of Liver and Kidney in Traditional Chinese Medicine, Wuhan, China; 5 Guangshui Traditional Chinese Medical Hospital, Suizhou, China

**Keywords:** ceRNA, circRNA, immune microenvironment, postmenopausal osteoporosis, senile osteoporosis

## Abstract

**Background:**

Osteoporosis (OP) pathogenesis is inextricably linked to immune microenvironment dysregulation. However, the sex-specific immunoregulatory disparities between senile osteoporosis (SOP) and postmenopausal osteoporosis (PMOP) remain incompletely elucidated.

**Methods:**

In this study, we integrated multi-cohort bulk transcriptomics, single-cell RNA sequencing (scRNA-seq), and independent clinical validations to systematically decipher the distinct immune landscapes and circular RNA (circRNA) regulatory axes underlying SOP and PMOP.

**Results:**

Bulk transcriptomic analysis revealed pervasive cytokine activation in OP, and such activation may be linked to divergent gene signatures between cohorts. Notably, *LAG3* and *TBX21* emerged as core cytokine-related hub genes across datasets. Immune deconvolution demonstrated sexually dimorphic microenvironment remodeling: male SOP patients exhibited a significant decrease in CD4^+^ T cells alongside expanded NK and CD8^+^ T cells, whereas female PMOP patients were primarily characterized by an increased proportion of monocytes. Furthermore, we constructed an immune-targeted competitive endogenous RNA (ceRNA) regulatory network and identified three core circRNAs (hsa-KMT5B_0001, hsa-TMEM55A_0001, and hsa-TNFRSF21_0001) as potential upstream regulators of these immune shifts. Subsequent scRNA-seq analysis of bone marrow mononuclear cells validated the peripheral immune imbalances and overexpressed the heightened expression of key cytokine genes predominantly to dendritic cells and monocytes. Finally, quantitative RT-PCR and flow cytometry in an independent clinical cohort further validated the upregulation of the target transcripts and the specific expansion of NK cells in SOP.

**Conclusion:**

This study unveils the sex-divergent immune-associated mechanisms underlying OP and delineates three novel circRNA-cytokine networks. These findings provide a theoretical basis and candidate molecular targets for the development of stratified, sex-specific clinical interventions for OP.

## 1 Introduction

Osteoporosis (OP) represents a major global public health burden characterized by compromised bone strength, microarchitectural deterioration, and an increased susceptibility to fragility fractures (1). Historically, OP has been highly prevalent among aging populations, encompassing two predominant clinical phenotypes: postmenopausal osteoporosis (PMOP) in females, primarily driven by estrogen withdrawal, and senile osteoporosis (SOP) in males, largely attributed to age-related physiological decline (2, 3). Although the fundamental pathology of both subtypes involves an uncoupling of bone remodeling—where osteoclast-mediated bone resorption outpaces osteoblast-mediated bone formation—the distinct upstream molecular triggers driving this imbalance across different sexes groups remain incompletely elucidated (4).

In recent years, the emerging field of “osteoclinical immunology” (or immunoporosis) has highlighted the inextricable link between the immune microenvironment and skeletal homeostasis (5, 6). Both innate and adaptive immune cells actively participate in bone metabolism by releasing a milieu of pro-inflammatory cytokines, such as interleukin-6 (IL-6) and tumor necrosis factor-alpha (TNF-α), which directly modulate osteoclastogenesis via signaling cascades like the nuclear factor kappa-B (NF-κB) pathway (5, 7). However, a critical gap exists in the current literature: most studies approach OP as a homogenous condition, overlooking the potential sexually dimorphic and cohort-specific immune trajectories. Deciphering whether SOP and PMOP are governed by distinct immune cell subpopulations and cytokine networks is vital for developing precise, patient-stratified therapeutic strategies (8, 9).

At the transcriptomic level, these complex immune responses and cytokine productions are tightly orchestrated by epigenetic regulators. Circular RNAs (circRNAs), a robust class of non-coding RNAs, have garnered significant attention for their role as competitive endogenous RNAs (ceRNAs) (10, 11). By acting as molecular “sponges” or decoys for microRNAs (miRNAs), circRNAs relieve the repressive effect on downstream target mRNAs, thereby serving as crucial upstream modulators of cellular function (11, 12). Increasing evidence suggests that specific circRNAs may dictate the activation, proliferation, and differentiation of immune cells, consequently altering the cytokine milieu in the bone microenvironment (13). Nevertheless, the comprehensive circRNA-miRNA-mRNA regulatory axes specifically governing the distinct immune landscapes of SOP and PMOP have not been systematically mapped.

To bridge this knowledge gap, we hypothesize that divergent circRNA-associated epigenetic networks correlate with the cohort-specific immune microenvironment remodeling observed in male SOP and female PMOP, and may participate in the pathological progression of OP. In this study, we systematically integrated and analyzed multi-cohort bulk RNA-sequencing and microarray datasets from both male and female OP patients to delineate their shared and unique cytokine profiles and immune cell infiltration patterns. Furthermore, we constructed an immune-targeted ceRNA network to identify core circRNA regulators. The robustness of our bioinformatic findings was subsequently validated through single-cell RNA sequencing (scRNA-seq) analysis of bone marrow mononuclear cells and empirical experimental validation (quantitative RT-PCR and flow cytometry) in an independent clinical cohort. This multidimensional approach aims to provide novel insights into the sex-specific pathogenesis of OP and identify diagnostic biomarkers and therapeutic targets.

## 2 Materials and methods

### 2.1 Study participants

Following screening based on strict inclusion and exclusion criteria, participants were recruited from three groups: healthy women, females with PMOP, and males with SOP. There were seven participants in each group.

Inclusion criteria:1)Aged 50–65 years; 2) Body mass index (BMI) ranging from 18.5 to 27.9 kg/m^2^; 3) HC group: healthy females with 4–6 years since natural menopause, and T-scores ≥ −1.0 at both lumbar spine and femoral neck; 4) SOP group: males with SOP, and T-scores ≤ −2.5 at both lumbar spine and femoral neck; 5) PMOP group: females with 4–6 years after natural menopause, and T-scores ≤ −2.5 at both lumbar spine and femoral neck.
Exclusion criteria:1) Use of medications affecting bone metabolism within six months prior to enrollment (bisphosphonates, parathyroid hormone analogs, selective estrogen receptor modulators, glucocorticoids, etc.); 2) History of previous fractures; 3) Presence of other acute infections or surgical procedures within four weeks before enrollment; 4) Comorbid active inflammatory diseases, severe cardiac, hepatic or renal failure, or malignant tumors.

### 2.2 Blood sample collection and processing

Peripheral blood (5–8 mL) was collected using sterile EDTA-K2 anticoagulant vacutainers. Tubes were gently inverted 5–8 times for homogenization and stored at 4 °C prior to quantitative RT-PCR and flow cytometry.

### 2.3 Quantitative reverse transcription polymerase chain reaction

Take 5 mL of whole blood and centrifuge it at 3000 rpm at room temperature for 5 minutes. Remove 200 μL of the supernatant and add 1 mL of Trizol reagent (Ambion, 15596026) to extract total RNA. Determine the concentration and purity of the RNA, and reverse-transcribe the RNA into cDNA using a kit (YEASEN, 11141ES60). Subsequently, perform quantitative RT-PCR amplification using a 20 μL reaction mixture under the following conditions: 3-minute pre-denaturation at 95 °C, 5-second denaturation at 95 °C, 10-minute annealing at 56 °C, and 25-second extension at 72 °C, repeated for 40 cycles. Then analyze the melting curve to determine whether the product has undergone specific amplification and analyze the amplification curve and calculate the Ct values. Using GAPDH as the internal reference gene, calculate the mRNA expression levels of the target genes. The primers were synthesized by Wuhan Tianyi Huayu Gene Technology, and the sequences are shown in **Table 1**.

**Table 1:** Primer sequence information.

Primer name	Sequence (5′-3′)	Size(bp)
*CX3CR1*	Forward primer:CAAGAAGCCCAAGAGTGT	155
	Reverse primer:TGAAGAAGAAGGCGGTAG	
*IL2RA*	Forward primer:CACGCCACATTCAAAGCC	270
	Reverse primer:TGGAAGGCTCGCTTGGTC	
*STAT1*	Forward primer:GCTTCTTGGTCCTAACGC	186
	Reverse primer:TCTCGCTCCTTGCTGATG	
*AIF1*	Forward primer:ATGCTGGAGAAACTTGGA	100
	Reverse primer:AGTCAGGGTAGCTGAACG	
*C3AR1*	Forward primer:GTTCCTCCACCTCACCTT	213
	Reverse primer:TGACACCAGATTGGCTTG	
*FCGR3A*	Forward primer:GGCAAAGGCAGGAAGTAT	186
	Reverse primer:CCCAGGTGGAAAGAATGA	
*SPON2*	Forward primer:TCGCTGGTCTCGTTTGTGG	212
	Reverse primer:GAGGAGGACGTTATCTCGGTCA	
*TBX21*	Forward primer:TCCCATTCCTGTCATTTACTGT	115
	Reverse primer:ACCCACTTGCCGCTCTG	
hsa-KMT5B_0001	Forward primer:GGTTGTATTGCCGAACTT	276
	Reverse primer:CTCGCAGAACTCATTATTTT	
hsa-TMEM55A_0001	Forward primer:CACATCTCGGCGAATAGG	258
	Reverse primer:TATGCACAGCAGCGTCTT	
hsa-TNFRSF21_0001	Forward primer:GTGGGCTGATGGAAGACA	181
	Reverse primer:TCCGACTCATCCACGAAG	
*GAPDH*	Forward primer:GGGAAACTGTGGCGTGAT	299
	Reverse primer:GAGTGGGTGTCGCTGTTGA	

### 2.4 Flow cytometric analysis of peripheral blood

Peripheral blood mononuclear cells (PBMCs) were isolated from 2 mL of whole blood using density gradient centrifugation. Resuspend the cell pellet in 100 μL of PBS, add 5 μL each of CD56 (eBioscience, 17-0567-41) and CD3 (eBioscience, MA1-10178) antibodies, and incubate at 4 °C in the dark for 30 minutes. Wash with 2 mL of PBS (SolarBio), centrifuge at 3000 rpm for 5 minutes, discard the supernatant, resuspend the cells in 200 μL of PBS, and finally measure the number of NK cells using a flow cytometer (Eisen, NovoCyte). Analyze the results using the NovoCyte software.

### 2.5 Statistical analysis

Statistical analyses in this study were performed using SPSS (version 27.0), while GraphPad Prism (version 10.0) was used for plotting graphs. Normally distributed quantitative data are expressed as mean ± standard deviation ( $\bar{x}\pm s$). One-way analysis of variance (ANOVA) was adopted for comparisons among multiple groups, followed by the least significant difference (LSD) test for pairwise comparisons. Non-normally distributed data are presented as median (interquartile range). The Mann–Whitney *U* test was used for comparisons between two groups, and the Kruskal–Wallis *H* test was applied for multiple-group comparisons. Spearman’s correlation analysis was performed for correlation assessment. Analysis of covariance (ANCOVA) was utilized to adjust for confounding factors. Categorical data are presented as case numbers (percentage) [*n* (%)], and intergroup comparisons were conducted using Fisher’s Exact Test. *P* < 0.05 was considered statistically significant.

### 2.6 Public data collection

**Figure 1:**
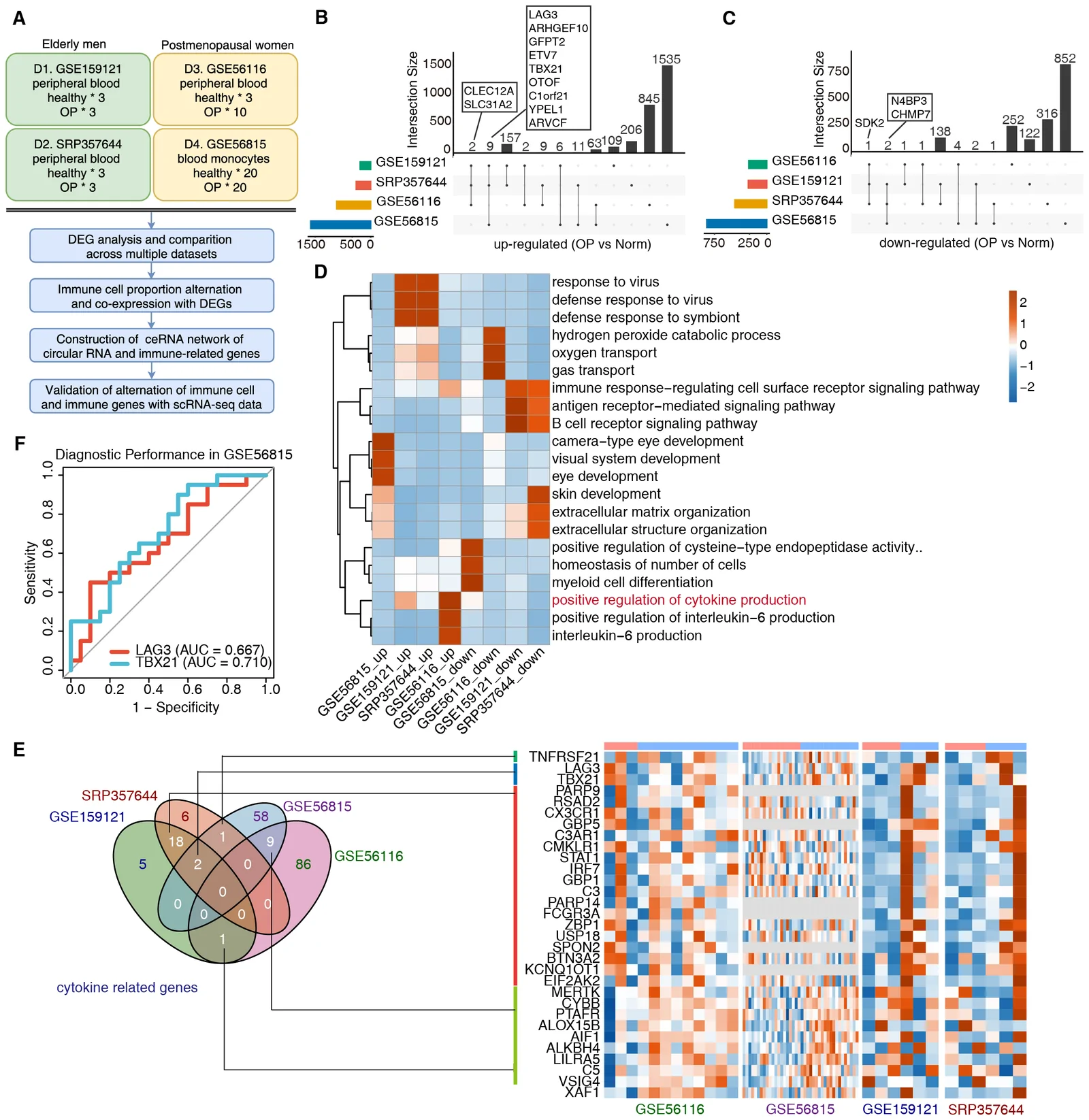
Pervasive cytokine activation characterized in osteoporotic patients. **(A)** Schematic illustration of the study design and analytical workflow. **(B, C)** Upset visualization of the intersection size of up-regulated **(B)** or down-regulated **(C)** genes comparing OP with healthy controls in different datasets. **(D)** GO biological processes enrichment of DEGs from different datasets. Top 3 terms were selected for each group and heatmap shows the enrichment q-value of these terms (scaled by column). **(E)** The Venn diagram in left part illustrates the overlap of up-regulated cytokine related genes identified in four datasets. The right part represents the expression profile of co-DEGs. **(F)** Receiver operating characteristic (ROC) curves demonstrating the diagnostic performance of *LAG3* and *TBX21* in distinguishing osteoporotic patients from healthy controls within the GSE56815 dataset.

The datasets related to elderly male OP, including GSE159121 and SRP357644, were acquired from the Sequence Read Archive (SRA). These datasets consist of peripheral blood samples from three individuals diagnosed with osteoporosis and three individuals classified as healthy. Additionally, data for postmenopausal women, including GSE56116 and GSE56815, were obtained from the GEO. GSE56116 comprises peripheral blood samples from ten individuals diagnosed with OP and three individuals classified as healthy, while GSE56815 includes blood monocyte samples from 20 individuals diagnosed with OP and 20 individuals classified as healthy. Single-cell transcriptional data from GSE147287 was also sourced from the GEO database. For detailed information regarding the datasets mentioned, please refer to **Figure 1A**.

### 2.7 RNA-seq data processing

For RNA-seq data, the raw RNA sequencing reads were first trimmed to remove adapters and low-quality bases by using fastp software (version 0.23.4) (14). All filtered reads were then aligned to the human reference genome (GRCh38) by HISAT2 (version 2.2.1) (15). For the rRNA-depletion dataset GSE159121, to meet the specific requirements of various circRNA identification tools, we employed three distinct mapping software tools: HISAT215(utilized for FindCirc16), STAR17 (used for circRNA_finder18 and CIRCexplorer219), and BWA20(applied for CIRI221). Uniquely mapped reads were ultimately used to calculate read number and reads per kilobase of exon per million fragments mapped (FPKM) for each gene. The expression levels of genes were evaluated using FPKM. When we do gene differential expression analysis, we choose the software DEseq2 (version 1.42.0) (16). Genes with *P ≤* 0.05 and fold change (*FC*) ≥1.5 (up) or ≤2/3 (down) and were considered as DEGs.

### 2.8 Microarray data processing

The expression profiles were converted and standardized by log2 to obtain a series matrix file. The LIMMA package was used to identify DEGs (17). Genes with *pvalue ≤* 0.05 and *FC* ≥1.5 (up) or ≤2/3 (down) were considered as DEG threshold values for DEG identification of GSE56116. Genes with *P ≤* 0.05 and *FC* ≥1.5 (up) or ≤2/3 (down) were considered as DEG threshold values for DEG identification of GSE56815.

### 2.9 ScRNA-seq data processing

The initial UMI count matrix underwent rigorous quality control (QC) and filtering using the R package Seurat (v5.0.2) (18). Cells failing to meet quality criteria, such as having UMI numbers < 500 or detected genes < 300 and > 8000, or exhibiting over 15% mitochondrial-derived UMI counts, were deemed low-quality and consequently excluded. Moreover, genes detected in less than 5 cells were also eliminated for downstream analyses. After quality control, the UMI count matrix was log normalized. Then top 2000 variable genes were used to create potential Anchors with FindIntegrationAnchors function of Seurat. Then, IntegrateData function was used to integrate data. Subsequently, to reduce dimensionality and mitigate potential batching effects, Principal Component Analysis (PCA) was executed.

Using the ‘Elbowplot’ function of Seurat, the top 50 principal components (PCs) were determined for downstream analysis. Subsequently, the primary cell clusters were identified through the ‘FindNeighbors’ (dims=1:50) and ‘FindClusters’ functions provided by Seurat (resolution = 0.4), resulting in the categorization of cells into 21 major cell clusters. The visualization of these cell clusters was achieved using UMAP (Uniform Manifold Approximation and Projection) with the ‘RunUMAP’ function (dims = 1:50). To characterize the clusters, marker genes were identified by employing the ‘FindAllMarkers’ function in Seurat, with criteria set at avg_log_2_*FC*≥0.5, pct.1-pct.2≥0.25, and p_val_adj ≤ 0.05. To ascertain the cell type for each cluster, we utilized a compilation of previously published marker genes (**Supplementary Figure 4A**). Initially, an automatic cell type annotation R package called ScType (19) was employed to annotate the cell types. Subsequently, a meticulous manual review and corrections were undertaken to refine the annotations. In the end, a total of 8 distinct cell types were successfully annotated.

### 2.10 Functional enrichment analysis

To identify functional categories of genes, we employed the clusterProfiler package (v4.6.2) (20), which enabled us to determine Gene Ontology (GO) terms and KEGG pathways.

### 2.11 Cell-type quantification

The CIBERSORT algorithm(v1.03) (21) along with the LM22 signature matrix was used with the default parameter for estimating immune cell fractions using FPKM values of each expressed gene. A total of 22 human immune cell phenotypes were analyzed in the study, including seven T cell types [CD8^+^ T cells, naïve CD4^+^ T cells, memory CD4^+^ resting T cells, memory CD4^+^ activated T cells, T follicular helper cells, and regulatory T cells (Tregs)]; naïve and memory B cells; plasma cells; resting and activated NK cells; monocytes; macrophages M0, M1, and M2; resting and activated dendritic cells; resting and activated mast cells; eosinophils; and neutrophils.

### 2.12 Co-expression analysis

The co-disturbed network between cell fraction of immune cells and expression of DEGs for each datasets was constructed. |Pearson’s correlation|≥0.9 and *P ≤*0.01 were retained for GSE159121, SRP357644, and GSE56116. |Pearson’s correlation| ≥0.6 and *Px ≤* 0.01 were retained for GSE56815.

### 2.13 CircRNA prediction and analysis

The identification of circRNAs for GSE159121 relies on the detection of “back-spliced reads” (BSJ). In this study, we employed four well-established circRNA prediction tools, namely FindCirc (22), circRNA_finder (23), CIRCexplorer2 (24), and CIRI2 (25), to predict circRNAs individually. Subsequently, we filtered and selected circRNAs predicted by at least two of these tools to constitute the final candidate circRNA dataset for downstream analysis. We integrated information from five well-established circRNA databases, namely circAtlas (26), circBase (27), circRNADb (28), deepbase2 (29), and circpedia2 (30). Predicted circRNAs were meticulously annotated based on this comprehensive database amalgamation. SRPBM (Spliced reads per billion mapped reads) (31) is used to represent the standardized expression of each circRNAs, that is, the number of back-spliced reads on each circRNAs in one billion reads. The formula is as follows: SRPBM (circRNA) = back-spliced reads(circRNA)*1000 000 000/total number of mapped reads. The R package TCC (version 1.40.0) (32) was applied to screen out the raw BSJ reads for detected differentially expressed circRNAs. The fold change (|log_2_*FC*| ≥ 1) and *P* ≤0.05 were set as the threshold to determine whether a circRNA was differentially expressed.

### 2.14 CeRNA network construction of circRNA, microRNA, and DEG

Given the known role of circRNAs as miRNA sponges, we employed two methods to predict target relationships between miRNAs and DE circRNAs. The first method involved Miranda (https://anaconda.org/bioconda/miranda) for predicting microRNA complementary pairing with circRNAs, retaining miRNA-circRNA pairings with a miRanda score of 160 or higher. The second method utilized Rnahybrid (https://bibiserv.cebitec.uni-bielefeld.de/rnahybrid/) to retain miRNA-circRNA pairs with a p-value less than or equal to 0.01. The final miRNA-circRNA target relationships were determined by taking the overlapping results from these two methods. To robustly support these computational predictions, we systematically queried the CircNet database for known circRNA-miRNA interactions. Subsequently, we integrated miRDB (http://mirdb.org) and TargetScan (http://www.targetscan.org) to cross-verify the target genes of the microRNAs. Ultimately, we constructed the ceRNA network comprising DE circRNA-miRNA-mRNA using Cytoscape software (v3.10.0, https://cytoscape.org/). In the network visualization, interactions supported by established databases were specifically highlighted.

### 2.15 Data integration and avoidance of batch effects

To rigorously mitigate technical batch effects and platform-specific biases inherent in utilizing multiple public datasets, an intersection-based meta-analysis strategy was employed in this study. Expression matrices from disparate cohorts (GSE159121, SRP357644, GSE56116, and GSE56815) were neither merged nor concatenated prior to analysis. Instead, normalization, differential expression gene (DEG) identification, and immune cell deconvolution were executed strictly and independently within each individual dataset. Subsequent cross-cohort integrations were restricted to the final analytical outputs (e.g., overlapping DEG lists and significant cellular shifts). By conducting comparisons exclusively at the results level, technical variances between sequencing platforms and microarray technologies were inherently prevented from propagating across cohorts, rendering mathematical cross-batch correction algorithms unnecessary.

### 2.16 Other analysis

Principal component analysis (PCA) was executed using the R package factoextra (https://cloud.r-project.org/package=factoextra) to illustrate sample clustering through the first two components. For clustering based on Euclidean distance, we employed the heatmap package (https://cran.r-project.org/web/packages/pheatmap/index.html) in R. Additionally, Receiver Operating Characteristic (ROC) curve analysis was performed, and the Area Under the Curve (AUC) was calculated to evaluate the diagnostic performance of the identified hub genes.

## 3 Results

### 3.1 Pervasive cytokine activation characterized in osteoporotic patients

To investigate individual differences in gene expression and function between SOP and PMOP, we analyzed peripheral blood RNA-sequencing and microarray datasets from the Gene Expression Omnibus (GEO) database, which were sourced from elderly males (GSE159121, SRP357644) and postmenopausal women (GSE56116, GSE56815) (**Figure 1A**). We identified 157 shared differentially expressed genes (DEGs) between two datasets of elderly males (adjusted *P* ≤ 0.05, |log_2_*FC*| ≥ 0.58), and 63 shared DEGs between two datasets of postmenopausal women (adjusted *P* ≤ 0.05, |log_2_*FC*| ≥ 0.58 or 0). Fewer shared DEGs were obtained among three or more groups, which indicates that the molecular mechanisms underlying SOP differ from those of PMOP (**Figure 1B**). Among the DEGs, *CLEC12A* and *SLC31A2* were found to be co-upregulated in both datasets of elderly male patients (GSE159121, SRP357644) and in the dataset of postmenopausal female patients (GSE56116) (adjusted *P* ≤ 0.05, log_2_*FC* ≥ 0.58). Additionally, 9 genes exhibited significant up-regulation in both the elderly male dataset (GSE159121, SRP357644) (adjusted *P* ≤ 0.05, log_2_*FC* ≥ 0.58) and another postmenopausal female dataset (GSE56815; adjusted *P* ≤ 0.05, log_2_*FC* ≥ 0), including *LAG3*, *ARHGEF10*, *GFPT2*, *ETV2*, *TBX21*, *OTOF*, *C1orf21*, *YPEL1*, and *ARVCF* (**Figure 1B**). Further, we analyzed the down-regulated genes across three datasets, which were *SDK2*, *N4BP3*, and *CHMP7* (adjusted *P* ≤ 0.05, log_2_*FC* ≥ 0.58) (**Figure 1C**). Furthermore, we conducted a functional analysis of the upregulated DEGs, and the results demonstrated that they were primarily enriched in pathways associated with cytokine production (**Figure 1D**). Then, we performed an intersection analysis of the cytokine - associated upregulated DEGs across the four datasets to identify their shared and unique expression patterns among the datasets. In particular, the genes *LAG3* and *TBX21* were found to be common in three of the datasets. Nine genes, including *MERTK*, *CYBB*, and *AIF1*, were commonly identified in data from postmenopausal women. Eighteen cytokine-related genes, such as *PARP9*, *CX3CR1*, *C3AR1*, *STAT1*, *FCGR3A*, and *SPON2*, were commonly detected in data from elderly men. Notably, the expression of these genes was found to be activated in OP samples (**Figure 1E**). These findings suggest that a widespread activation pattern is observed in both elderly male and postmenopausal female patients with OP, although the activated cytokine genes vary. Furthermore, to rigorously evaluate the diagnostic and translational potential of the identified core biomarkers, we performed ROC curve analysis for the shared hub genes, *LAG3* and *TBX21*. For this specific evaluation, we intentionally selected the GSE56815 dataset, as it provides the largest and most balanced sample size among the included transcriptomic cohorts, thereby ensuring adequate statistical power. Both genes exhibited excellent predictive capability in distinguishing osteoporotic patients from healthy controls (**Figure 1F**).

### 3.2 Elderly male osteoporosis patients exhibit a decrease in CD4^+^ T cell ratio and an increase in NK and CD8^+^ T cell ratios, whereas postmenopausal female patients show an increased ratio of monocytes

**Figure 2:**
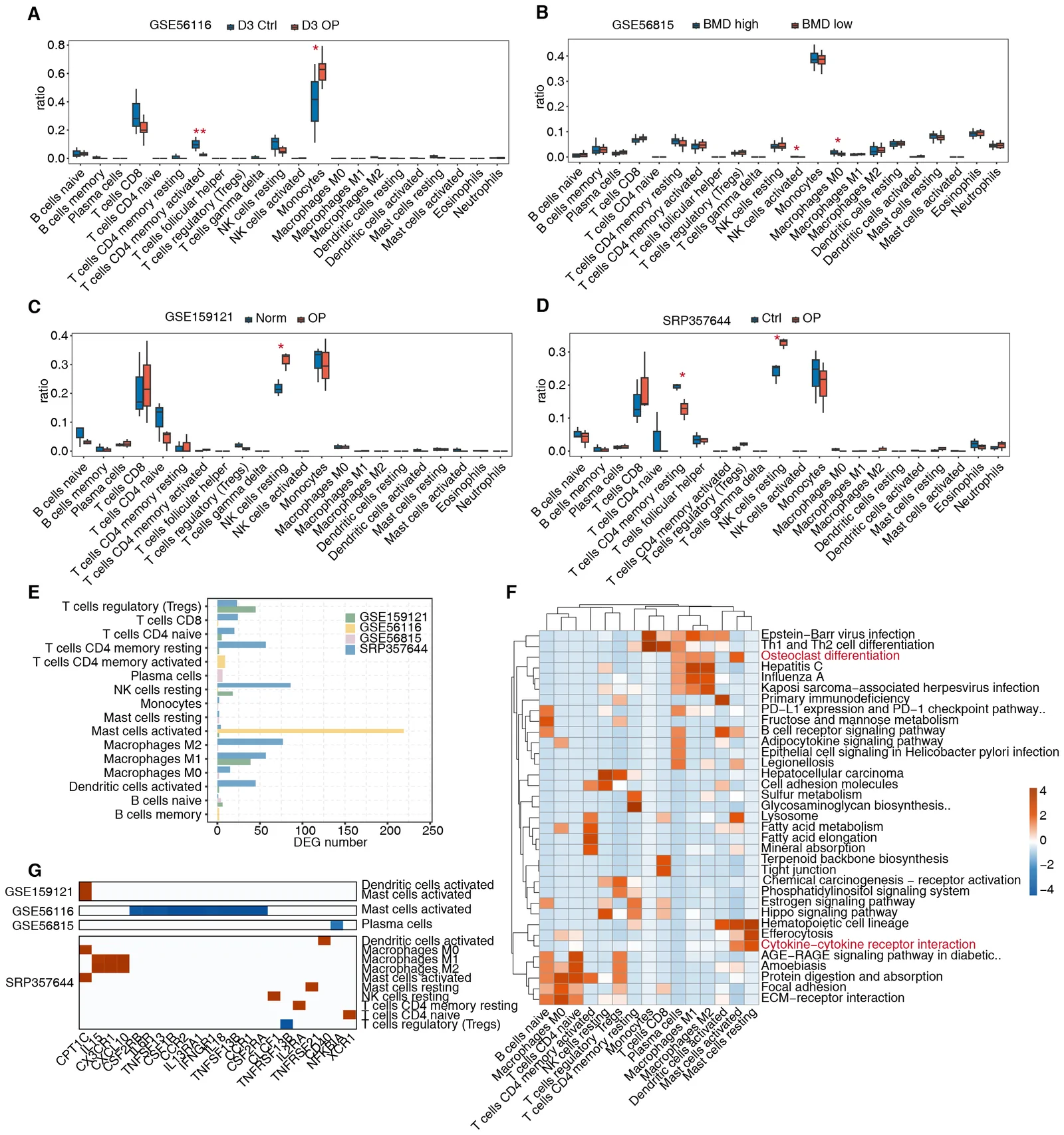
Elderly male osteoporosis patients exhibit a decrease in CD4^+^ T cell ratio and an increase in NK and CD8^+^ T cell ratios, whereas postmenopausal female patients show an increased ratio of monocytes. **(A–D)** Boxplot showing the fraction of each immune cell type in normal or OP samples of four datasets; the significant difference in the immune cell fractions was calculated using the student’s t test. *P ≤ 0.05, **P ≤ 0.01. **(E)** Bar plot shows the number of co-expressed DEGs with immune cell proportions in four datasets. **(F)** GO biological processes enrichment of cell type co-expressed DEGs. Top 3 terms were selected for each cell type and heatmap shows the enrichment q-value of these terms (scaled by column). **(G)** Association of cell infiltration and cytokine related gene expression in four datasets. Red color indicates positive correlation and blue indicate negative correlation.

To comprehensively evaluate the immune cell characteristics of OP across different populations, we employed the CIBERSORT algorithm to analyze the standardized gene expression matrices, thereby determining the proportional changes of various immune cell types across the four datasets (**Figures 2A–D**). In the dataset GSE56116 of postmenopausal women with OP, activated memory CD4^+^ T cells were significantly down-regulated and monocytes significantly up-regulated in OP samples compared with the control group(*P* < 0.05) (**Figure 2A**). The dataset GSE56815 contains data on monocytes, and the data indicates that the main components of monocytes in this dataset are consistent with expectations. (*P* < 0.05) (**Figure 2B**).In two datasets of elderly male patients with OP, the proportion of resting NK cells was significantly increased, while the proportion of CD4^+^ T cells was decreased and that of CD8^+^ T cells was increased in OP samples compared with normal controls (all *P* < 0.05) (**Figures 2C, D**). Further, we identified DEGs significantly associated with distinct immune cell types by correlating DEG expression levels with immune cell proportions across four datasets (Pearson *r* ≥ 0.9 or 0.6, *P* ≤ 0.01) (**Figure 2E**). KEGG pathway enrichment analysis was performed on DEGs co-expressed with immune cells, with a focus on biological pathways involved in osteoclast differentiation and cytokine-cytokine receptor interactions (**Figure 2F**). Next, we respectively showed the correlations of immune-related genes (**Supplementary Figure 2E**), osteoclast differentiation-related genes (**Supplementary Figure 2F**), and cytokine-related genes **Figure 2G**) with distinct immune cell subsets. Among these, we found that T cell-associated genes such as *NFKBIA*, *XCR1* and *IL2RA*, as well as NK cell-associated genes including *CLCF1*, exhibited strong correlations with their respective immune cell types (Pearson r ≥ 0.9 or 0.6, *P* ≤ 0.01), warranting further attention.

### 3.3 Construction of ceRNA network of circRNA and immune-related genes in osteoporosis

**Figure 3:**
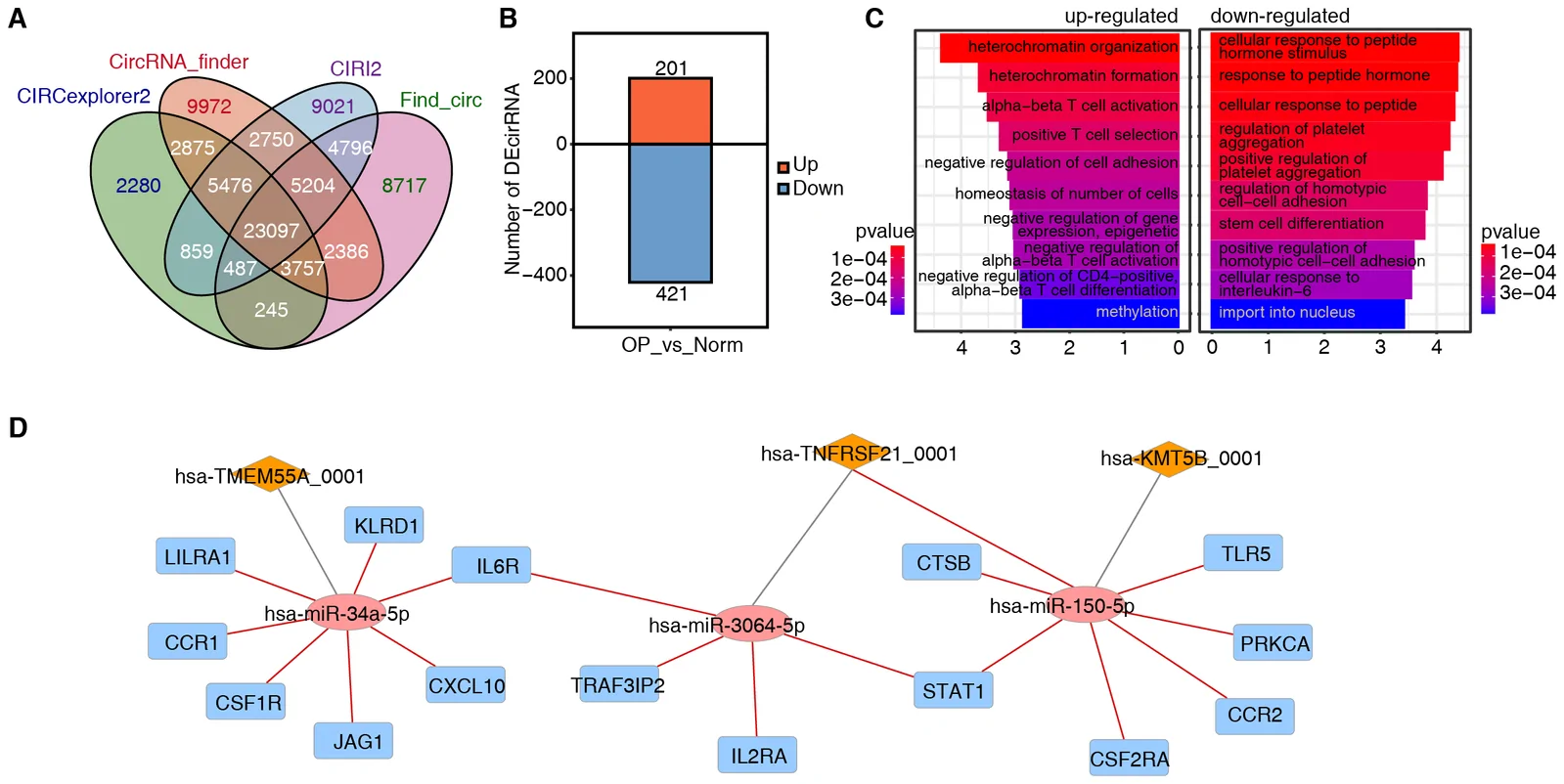
Construction of ceRNA network of circRNA and immune-related cytokine genes in osteoporosis. **(A)** Four widely used circRNA prediction tools (FindCirc, circRNA_finder, CIRCexplorer2, and CIRI2) to predict circRNA respectively, and then circRNAs predicted by at least two tools as the final candidate circRNA for downstream analysis. **(B)** Bar plot showing the 201 up-regulated circRNAs and 421 down-regulated circRNAs comparing OP with normal samples. **(C)** Bar plot showing the most enriched GO biological process results of host genes of up regulated and down regulated circRNAs in OP group. **(D)** The ceRNA network was established using DE circRNAs, miRNAs, and immune-related genes. Predictions of miRNA-circRNA target relationships were made using both Miranda and Rnahybrid. Additionally, miRNA-mRNA target pairs were obtained from the miRDB (http://mirdb.org) and TargetScan (http://www.targetscan.org) databases to predict the target relationship between miRNAs and mRNAs. Red edges represent interactions supported by evidence from public interaction databases (CircNet, TargetScan, or miRDB), whereas grey edges represent computationally predicted interactions.

To investigate upstream circRNAs that may regulate differentially expressed genes in OP and to construct a ceRNA network, we performed RNA sequencing on male peripheral blood samples from the GSE159121 dataset using an rRNA-depleted library preparation. Four circRNA prediction tools (FindCirc, circRNA_finder, CIRCexplorer2, CIRI2) were applied to minimize false-positive rates. circRNAs detected by at least two tools were considered reliable, yielding 28,835 circRNAs for downstream analysis (**Figure 3A**). Detailed characterization of these circRNAs is provided in the **Supplementary Materials** (**Supplementary Figures 3A–E**). Subsequently, we analyzed differentially expressed circRNAs and found that the SOP group exhibited 201 upregulated and 421 downregulated circRNAs (|log_2_*FC*| ≥ 1 and *p ≤* 0.05) (**Figure 3B**; **Supplementary Figure 3F**). Functional enrichment analysis of the host genes corresponding to these differentially expressed circRNAs revealed that upregulated host genes were primarily enriched in T-cell-related biological pathways, whereas downregulated host genes were predominantly enriched in cell-cell adhesion-related pathways (**Figure 3C**). Our study further identified potential upstream circRNA regulators of immune-related genes and constructed a circRNA-miRNA-mRNA network map corresponding to these immune-related genes (**Figure 3D**; **Supplementary Figure 2E**). To overcome the statistical limitations of our small sample size in direct expression correlation analysis, we validated a substantial proportion of these proposed pairings using publicly available interaction databases. In the network diagram, database-supported interactions are highlighted with red edges, while purely computational predictions are denoted by grey edges. We identified three circRNAs: hsa-KMT5B_0001, hsa-TMEM55A_0001, and hsa-TNFRSF21_0001, which suggests their potential involvement in OP development by regulating the expression of immune-related genes.

### 3.4 Single-cell data validate the changes in immune cell proportions and the upregulation of cytokine gene expression in osteoporotic patients

**Figure 4:**
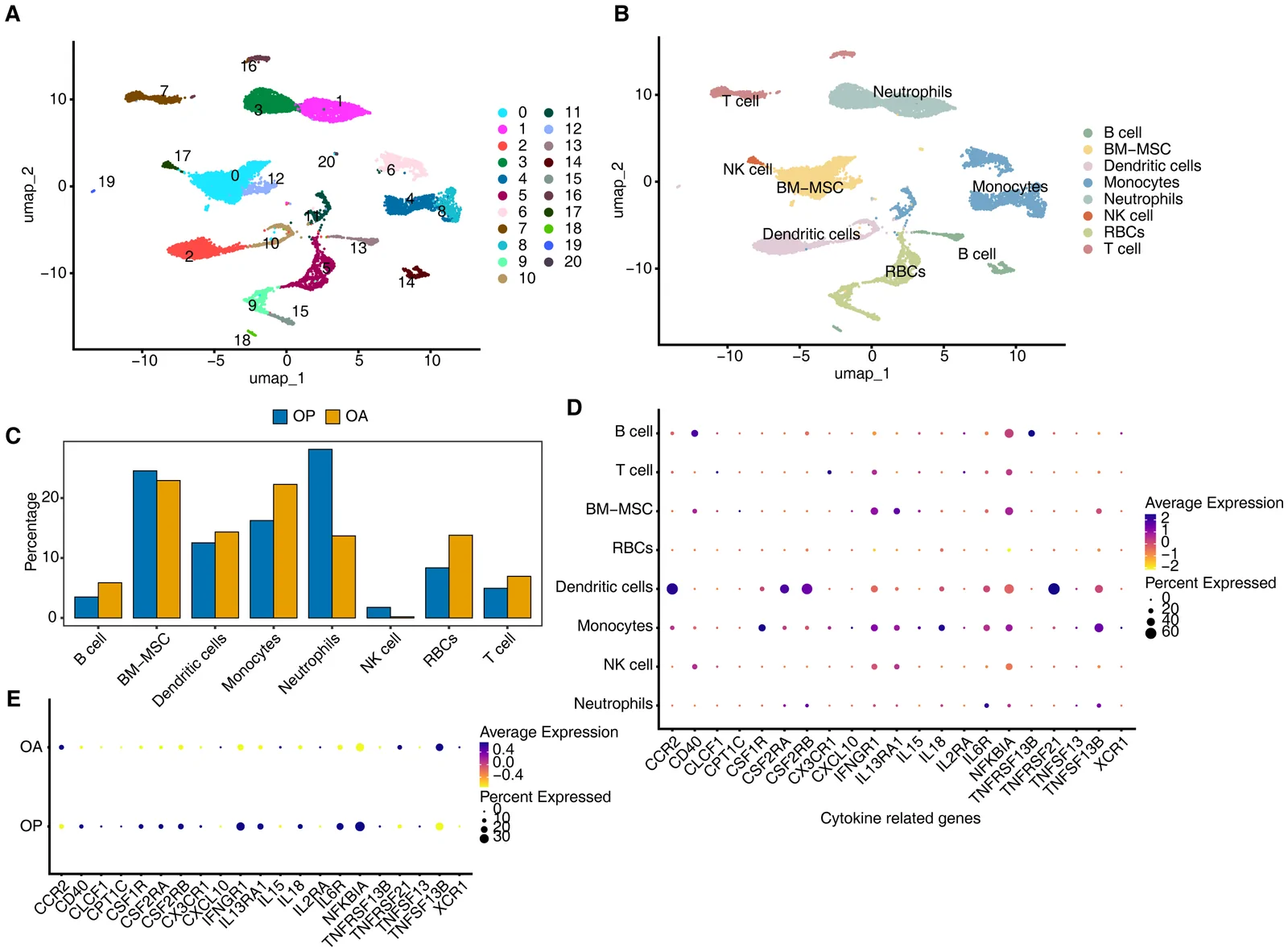
Single-cell data validate the changes in immune cell proportions and the upregulation of cytokine gene expression in osteoporotic patients. **(A)** Uniform Manifold Approximation and Projection (UMAP) lot of composite single-cell transcriptomic profiles. Colors indicate cell clusters. **(B)** UMAP plot of composite single-cell transcriptomic profiles from all samples. Colors indicate annotated cell types. **(C)** Bar plot comparing the relative proportions of each annotated immune cell cluster between the OP and OA groups. **(D)** Dot plot showing expression patterns of cytokine related genes in each cell type. Dot size denotes the percentage of cells expressing the corresponding gene and dot color represents the average expression. **(E)** Dot plot showing expression patterns of cytokine related genes in OP and OA samples. Dot size denotes the percentage of cells expressing the corresponding gene and dot color represents the average expression.

The bioinformatics analyses described above were all performed on peripheral blood samples and systematically identified a series of cytokine and immune cell abnormalities. However, given that the bone marrow microenvironment is a critical site for OP pathogenesis, whether similar abnormalities exist in bone marrow cytokines and immune cells remains a question. Moreover, since most immune cells in peripheral blood originate from the bone marrow and can migrate back to it under specific signaling cues, we proceeded to analyze transcriptomic data from bone marrow-derived monocytes (GSE147287) by RNA-sequencing. After rigorous data quality control and standardization, principal component analysis (PCA) was performed, and the top 50 principal components were selected for UMAP dimensionality reduction and visualization. Unbiased clustering analysis identified 21 cell subclusters (**Figure 4A**) and classified eight major immune cell types (**Figure 4B**; **Supplementary Figure 4A**), including neutrophils, monocytes, dendritic cells, NK cells, and T cells. Consistent with previous findings, we observed an increase in NK cells and a decrease in T and B cells in the OP group (**Figure 4C**). To investigate the expression profiles of cytokine-related genes across different cell populations, we examined their average expression levels. These genes were predominantly expressed in dendritic cells and monocytes. For instance, *CCR2* and *CSF2RA* were predominantly highly expressed in dendritic cells (**Figure 4D**). Moreover, the expression levels of these cytokine-related genes were significantly higher in the OP group than in the control group (**Figure 4E**). Genes involved in osteoclast differentiation, such as *STAT2*, *IFNGR1*, and *NFKBIA*, were significantly expressed in bone marrow mesenchymal stem cells, dendritic cells, monocytes, and NK cells (**Supplementary Figure 4B**), and their expression was also higher in the OP group (**Supplementary Figure 4C**).

### 3.5 Comparison of parameters among healthy women, females with postmenopausal osteoporosis, and males with senile osteoporosis

#### 3.5.1 Baseline characteristics of the three groups

To further explore the clinical relevance of the above findings, peripheral blood samples were collected from healthy women, females with PMOP and males with SOP, and quantitative RT-PCR was performed to detect the expression levels of eight cytokine-related genes and three circRNAs. Differential analyses of baseline data revealed no statistically significant differences in age, years since menopause, BMI, drinking history, smoking history, history of hypertension, history of coronary heart disease, and medication history between the HC group and the PMOP group (*P*>0.05). Significant intergroup differences were observed in serum 25-hydroxyvitamin D [25(OH)D] levels, lumbar spine and femoral neck bone mineral density, as well as T-scores between the two groups (*P* < 0.05). No significant differences in baseline characteristics were detected between the PMOP group and the SOP group (*P*>0.05) (**Table 2**).

**Table 2:** Comparison of clinical characteristics among the three study groups [( $\bar{x}$ **±** s), *M*(*P*_25_,*P*_75_), n(%)].

Variables	HC(*n* = 7)	PMOP(*n* = 7)	SOP(*n* = 7)	*F*/*U*/*H*	*P*
Age	56.43 ± 3.10	56.86 ± 2.41	57.00 ± 2.16	0.09	0.912
Menopausal duration	4(4,5)	5(4,6)	-	20.00	0.530
BMI(kg/m2)	22.91 ± 0.80	23.55 ± 1.27	23.74 ± 1.51	0.89	0.429
Disease duration(year)	-	4(3,5)	5(4,5)	20.50	0.595
Alcohol consumption	1(14.29)	2(28.57)	4(57.14)	-	0.381
Smoking status	2(28.57)	1(14.29)	3(42.86)	-	0.829
Hypertension	1(14.29)	2(28.57)	3(42.86)	-	0.829
CHD	0(0.00)	2(28.57)	1(14.29)	-	0.742
Medication	4(57.14)	3(42.86)	2(28.57)	-	0.854
25 (OH) D (ng/mL)	24.11 ± 4.67	19.50 ± 2.32^*^	19.21 ± 4.15	3.58	0.049
BMD (Lumbar spine)/(g/cm^2^)	0.91(0.89,0.94)	0.63(0.605,0.655)^***^	0.655(0.636,0.694)	14.83	<0.001
BMD(Femoral neck)/(g/cm^2^)	0.80(0.79,0.83)	0.536(0.5,0.559)^***^	0.53(0.522,0.574)	13.52	<0.001
T-score (Lumbar spine)	-0.77(-0.92,-0.5)	-3(-3.2,-2.8)^***^	-3.1(-3.24,-2.8)	13.38	<0.001
T-score (Femoral neck)	-0.6(-0.75,-0.38)	-2.9(-3.21,-2.7)^***^	-2.94(-3,-2.6)	13.52	<0.001

History of medication use includes antihypertensives, hypoglycemic agents, vitamin D, calcium supplements.

Compared with the HC: *P < 0.05, ***P < 0.001.

#### 3.5.2 Clinical detection of mRNAs, circRNAs and NK cells

**Figure 5:**
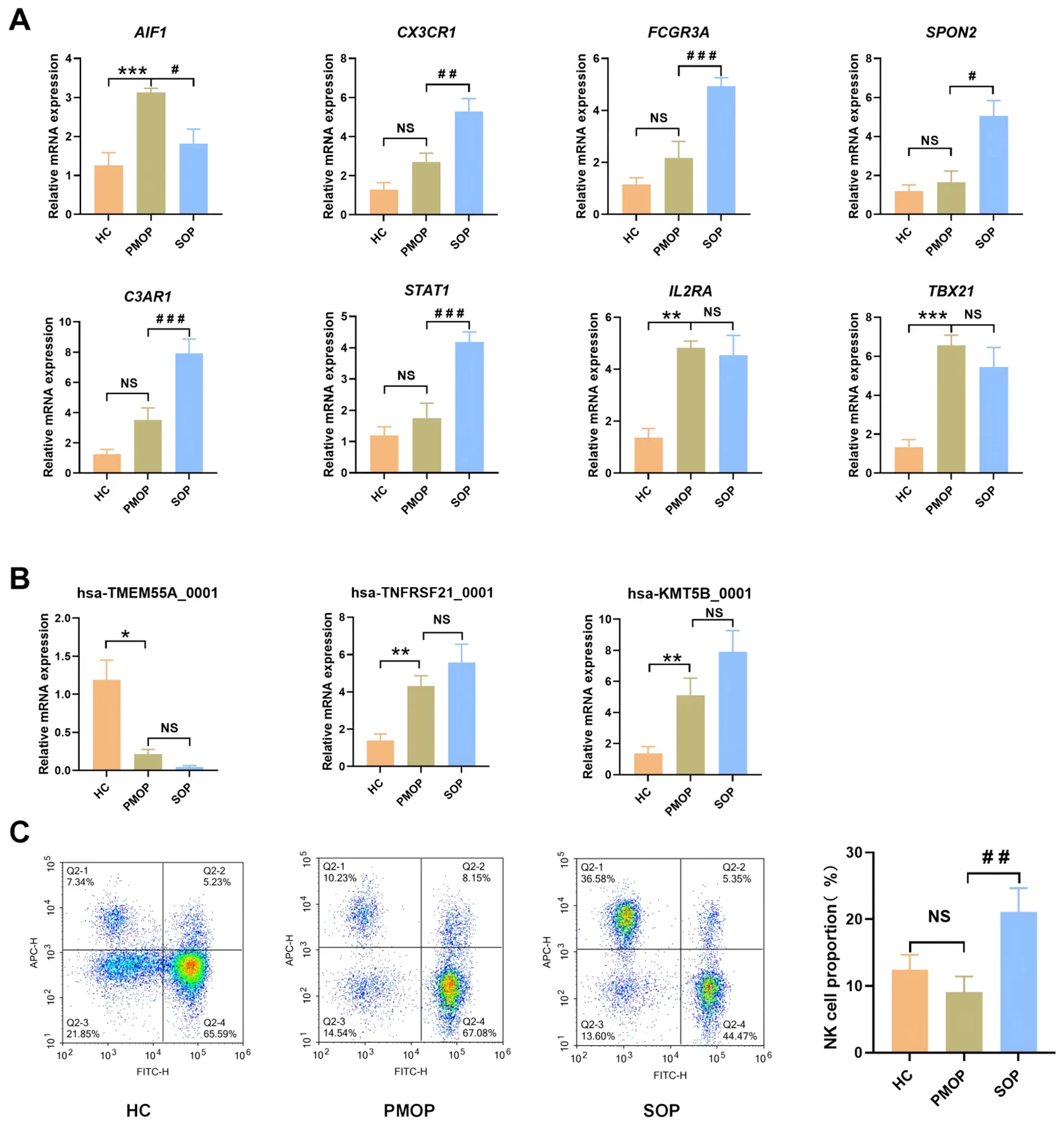
Clinical detection of mRNAs, circRNAs and NK cells. **(A)** Relative expression levels of differential genes in peripheral blood from different populations. **(B)** Relative expression levels of circRNA in peripheral blood from different populations. **(C)** Proportion of NK cells in peripheral blood from different populations. **(A–C)** Compared with the HC: ^*^P < 0.05, ^**^P < 0.01, ^***^P < 0.001; Compared with the PMOP: ^#^*P* < 0.05, ^##^*P* < 0.01,^###^*P* < 0.001; No significant difference (NS): *P*>0.05.

After adjusting for confounding factors, analyses demonstrated that compared with the HC group, the expression levels of *AIF1*, *IL2RA*, *TBX21*, hsa-KMT5B_0001 and hsa-TNFRSF21_0001 were significantly upregulated in patients with PMOP, whereas hsa-TMEM55A_0001 was markedly downregulated (all *P* < 0.05). Compared with the PMOP group, the SOP group exhibited elevated expression of *CX3CR1*, *STAT1*, *C3AR1*, *FCGR3A* and *SPON2*, accompanied by decreased *AIF1* expression (all *P* < 0.05). In addition, flow cytometry was used to compare the proportion of peripheral blood NK cells across groups. The proportion of peripheral blood NK cells was significantly higher in the SOP group relative to the PMOP group (*P* < 0.05), while no significant difference was found between the PMOP group and the HC group (*P* > 0.05) (**Figures 5A–C**; **Supplementary Table S1**).

#### 3.5.3 Correlation validation of the ceRNA network

To further verify the correlations within the ceRNA network, Spearman’s correlation analysis was conducted to evaluate the associations between differentially expressed genes and circRNAs. The results indicated that hsa-TNFRSF21_0001 was correlated with *IL2RA* (*r* = 0.72, *P* < 0.001) and *STAT1* (*r* = 0.52, *P* < 0.05). A significant correlation was also identified between hsa-KMT5B_0001 and *STAT1* (*r* = 0.53, *P* < 0.05).

## 4 Discussion

Under physiological conditions, bones undergo continuous remodeling, and skeletal homeostasis depends on the balance between osteoblast and osteoclast functions (33). However, this process is not intrinsic to bone tissue but is influenced by signals from immune cells and the skeletal microenvironment (34). The present study revealed significant sex-related heterogeneity in OP through differential gene expression analysis. Across four datasets, only 63 genes were upregulated and 4 downregulated in female patients with PMOP, whereas male patients with SOP exhibited more pronounced expression changes, with 157 genes upregulated and 138 downregulated. At the functional level, this difference may reflect distinct pathogenic mechanisms—estrogen deficiency versus inflammatory aging—in the process of bone loss mediated by immune remodeling (35, 36). Genes specifically highly expressed in female PMOP patients, such as *AIF1*, are more strongly associated with activation of the mononuclear-phagocyte system and osteoclast differentiation, suggesting that estrogen withdrawal may lead to bone loss through enhanced myeloid-cell-mediated bone resorption (37, 38). For instance, AIF1 is produced by monocytes, macrophages and lymphocytes, and its overexpression directly promotes the proliferation and migratory capacity of osteoclast precursors (35). In contrast, the DEGs specifically upregulated in male SOP populations (*CX3CR1* (37), *STAT1* (38), *FCGR3A* (39), *SPON2* (40), *C3AR1* (41) are predominantly enriched in pathways such as T-cell activation and interferon responses, consistent with the features of lymphocyte depletion and compensatory activation induced by inflammatory aging. This suggests that gene expression alterations in male SOP are more closely associated with age-related inflammatory responses and immune regulation (42, 43). For example, *CX3CR1* amplifies inflammatory responses via the extracellular signal-regulated kinase/mitogen-activated protein kinase (ERK/MAPK) signaling pathway, thereby exacerbating bone tissue damage37; *STAT1* serves as a key mediator in the type I interferon-α/β (IFN-α/β) signaling pathway, and its deficiency activates Runx2 to promote osteogenic differentiation (38); *FCGR3A* is involved in macrophage-mediated bone resorption (39); SPON2 facilitates dendritic cell maturation and T-cell activation while disrupting bone microstructure (40); and C3aR (encoded by *C3AR1*) has been identified as a critical target in age-related bone loss, with experimental evidence showing that C3aR knockdown inhibits osteoblast precursor cell differentiation through activation of the YAP1/β-catenin signaling pathway (41). Although sex-specific DEGs exhibit considerable variation, two core shared genes—*LAG3* and *TBX21*—are consistently identified, and demonstrates good predictive performance for OP (**Figures 1E, F**).As an immunosuppressive co-receptor, *LAG3* mediates regulatory T cell (Treg) metabolism to maintain immune homeostasis; however, its overexpression inhibits CD4^+^ T cell proliferation and reduces their ability to secrete interferon-γ (IFN-γ), thereby accelerating bone damage (44, 45). *TBX21* is a well-characterized pivotal gene associated with OP and participates in bone metabolic imbalance by regulating T cell differentiation (46). Multiple studies have demonstrated that a chronic, low-grade, non-infectious systemic inflammatory state is prevalent in both female PMOP patients and male SOP patients, characterized by elevated peripheral blood neutrophil-to-lymphocyte ratio, platelet-to-lymphocyte ratio, and systemic inflammatory markers (43, 47, 48). This suggests that regardless of the underlying etiology, bone metabolic imbalance ultimately leads to immune exhaustion. Subsequent clinical quantitative RT-PCR analyses have further validated the expression profiles of these genes, providing evidence to distinguish the pathogenic mechanisms of SOP and PMOP.

Immune cell subpopulation imbalance is a key pathological feature of OP33. The differential immune cell profiles revealed in this study further support and elucidate the aforementioned sex-specific differences: male SOP patients exhibit reduced proportions of CD4^+^ T cells, accompanied by increased proportions of NK cells and CD8^+^ T cells. Under normal conditions, NK cells and CD8^+^ T cells secrete bone-protective factors such as IFN-γ to mitigate bone resorption (34). With aging, their protective effects on bone tissue gradually diminish, and they may even acquire an inflammatory phenotype by secreting factors such as TNF-α and RANKL, thereby disrupting the bone microenvironment (49–52). In contrast, CD4^+^ T cells produce anti-inflammatory factors including IL-10 to suppress osteoclast activation (53). Female PMOP patients, however, show elevated monocyte proportions (**Figures 2A-D**). As precursors of osteoclasts, increased monocyte levels may directly contribute to enhanced bone resorption (54, 55). These findings suggest that both male SOP and female PMOP share a common pathological hallmark—dysregulation of the lymphoid versus myeloid cell balance—which is crucial for understanding their distinct immunopathological mechanisms. Notably, the immune cell lineage, activation status, and functional regulatory networks differ between human peripheral blood and bone marrow. Circulating immune cells in peripheral blood mainly reflect systemic immune and inflammatory status (56). As the primary site of hematopoiesis and immune cell development, bone marrow exhibits pronounced tissue specificity with respect to immune cell composition, differentiation stages, and functional regulation. In the present study, both single-cell RNA sequencing of bone marrow-derived cells and flow cytometric analysis of peripheral blood revealed a higher proportion of NK cells in males with SOP (57). Although the findings obtained from these two tissue compartments cannot be directly compared equivalently, they preliminarily indicate the correlative tendency between systemic and local immune dysregulation in patients with OP. Accordingly, therapeutic strategies for OP should place greater emphasis on restoring the balance of lymphocyte subsets rather than relying merely on hormone supplementation. The sex-specific characteristics of these immune cell subpopulations suggest that male SOP and female PMOP may be driven by distinct upstream molecular mechanisms. However, the epigenetic regulatory mechanisms governing the differentiation of these immune cell lineages, particularly the regulatory networks at the non-coding RNA level, remain poorly understood.

CircRNAs can regulate the activation, proliferation, and differentiation of immune cells by sponging miRNAs, thereby reshaping cytokine networks (12). Therefore, systematic identification of differentially expressed circRNAs in OP and elucidation of their ceRNA regulatory networks may uncover the molecular mechanisms underlying immune microenvironment dysregulation at the post-transcriptional level. Using multiple detection methods, we identified differentially expressed circRNAs and performed functional enrichment analysis, revealing that the corresponding upregulated host genes were predominantly enriched in T-cell-related biological pathways (**Figure 3C**). Subsequently, we constructed ceRNA networks for three core differentially expressed circRNAs and identified three specific circRNAs—hsa-TMEM55A_0001, hsa-TNFRSF21_0001, and hsa-KMT5B_0001—which may play pivotal roles in bone metabolism.

First, results from the ceRNA network analysis and Spearman’s correlation test revealed that upregulated hsa-TNFRSF21_0001 was significantly correlated with overexpressed *IL2RA* and *STAT1*. Previous studies have shown that *TNFRSF21* can induce T cell apoptosis (58, 59), whereas *IL2RA* is a critical receptor for T cell activation and maintenance of regulatory T cell function (60). The present study also confirmed an association between *IL2RA* and T cells (**Figure 2G**). Meanwhile, we identified imbalanced proportions of CD4^+^ T and CD8^+^ T cells in patients with OP (**Figure 2D**). *STAT1* also merits attention, which is involved in this network. *STAT1* not only enhances the cytotoxic activity of NK cells and CD8^+^ T cells but also directly inhibits osteogenic differentiation (61–63).Thus, we postulate that this signaling network correlates with impaired systemic lymphocyte homeostasis in OP patients.

Second, the upregulated hsa-KMT5B_0001 was significantly correlated with *STAT1*, *CTSB*, and *CCR2*, as well as *CSF2RA*. Previous studies have shown that elevated *KMT5B* expression correlates with reduced expression of bone formation-related proteins such as RUNX2, DSPP, and OPN, whereas targeted downregulation of *KMT5B* significantly increases mineralization in dental pulp stem cells (64, 65). In addition, miR-150-5p has been demonstrated to suppress *STAT1* expression and thereby mitigate inflammatory responses (38). The functions of its downstream target genes *CTSB* and *CSF2RA* should not be overlooked: *CTSB* participates in bone resorption by degrading bone matrix proteins (66); *CSF2RA* mediates GM-CSF-induced proliferation and differentiation and promotes the formation of inflammatory osteoclast precursors (67). Our previous single-cell sequencing studies also revealed that *CTSB* and *CSF2RA* are closely associated with dendritic cells, which have been shown to generate osteoclasts more efficiently than monocytes (68). Therefore, the hsa-KMT5B_0001/miR-150-5p axis may provide a valuable reference for understanding the imbalance between bone formation and resorption.

Third, quantitative RT-PCR results showed downregulated hsa-TMEM55A_0001 was correlated with genes including *CCR1* and *CXCL10* (**Figure 5B**). *CCR1* and *CXCL10* respectively mediate the chemotactic recruitment of osteoclast precursors and immune cells to the bone surface via their cognate receptors (69, 70). Correlation analyses further confirmed a close relationship between *CXCL10* and macrophages (**Figure 2G**). Additionally, *TMEM55A* exhibits phosphatase activity that is regulated by redox status (71), and excessive accumulation of reactive oxygen species in the OP bone microenvironment may inhibit its catalytic activity, thereby disrupting the PI5P-F-actin signaling pathway and exacerbating osteoblastic dysfunction (40, 72, 73). Thus, this axis provides a reference for understanding the abnormalities in the bone microenvironment of OP patients.

Integrating these three axes, upregulated hsa-TNFRSF21_0001 and hsa-KMT5B_0001 exhibit enhanced binding to miR-3064-5p and miR-150-5p, which correlates with elevated expression of *IL2RA* and *STAT1*, increased proliferation of cytotoxic lymphocytes, and suppressed bone formation (60, 61). In contrast, downregulated hsa-TMEM55A_0001 facilitates the release of miR-34-5p and is associated with reduced immune cell migration toward bone tissue (69, 70). Collectively, these three axes offer crucial insights into the pathogenesis of OP and facilitate the identification of novel targets for bone immune regulation.

In summary, this study identified differential gene targets, immune cell characteristics, and ceRNA networks that distinguish SOP and PMOP patients, offering a new perspective on the molecular mechanisms underlying OP. Nevertheless, several limitations should be acknowledged. First, the relatively small sample size may limit the statistical power and generalizability of the findings, as well as the statistical significance of direct expression correlation analyses within specific cohorts. Future studies with larger cohorts are needed to validate potential therapeutic targets. Second, although we utilized public interaction databases to cross-verify our networks and performed quantitative RT-PCR to confirm expression dysregulation *in vivo*, the regulatory functions of the identified circRNAs are currently based on bioinformatic predictions and clinical correlations, which limits direct causal interpretation. Functional validation of their sponge effect using luciferase reporter assays or RNA pulldown experiments is required to fully elucidate the physical binding mechanisms and role of circRNAs in OP and to explore their clinical potential.

## Data Availability

The datasets related to elderly male OP, including GSE159121 and SRP357644, were acquired from the Sequence Read Archive (SRA). These datasets consist of peripheral blood samples from three individuals diagnosed with osteoporosis and three individuals classified as healthy. Additionally, data for postmenopausal women, including GSE56116 and GSE56815, were obtained from the GEO. GSE56116 comprises peripheral blood samples from ten individuals diagnosed with OP and three individuals classified as healthy, while GSE56815 includes blood monocyte samples from 20 individuals diagnosed with OP and 20 individuals classified as healthy. Single-cell transcriptional data from GSE147287 was also sourced from the GEO database. For detailed information regarding the datasets mentioned, please refer to **Figure 1A**.
