# Supplementary material for: Elevated cytokines in osteoporotic blood correlate with immune microenvironment shifts and dysregulated circRNA profiles
Source: Front Immunol. 2026 Sep 9;17:1914272. doi: 10.3389/fimmu.2026.1914272 (PMC13597400; doi:10.3389/fimmu.2026.1914272)
Supplement: Supplementary file 1 [file SupplementaryFile1.docx]

Supplementary Material

## 1. Supplementary Figures

**Supplementary Figure 1.** **Pervasive Cytokine Activation Characterized in Osteoporotic Patients.**

Heatmap showing the expression profile of DEGs of four datasets. B. Principal component analysis (PCA) of expression level (FPKM) of DEGs of four datasets. The samples were grouped by OP or normal and the ellipse for each group is the confidence ellipse.

**Supplementary Figure 2. Elderly male osteoporosis patients exhibit a decrease in CD4+ T cell ratio and an increase in NK and CD8+ T cell ratios, whereas postmenopausal female patients show an increased ratio of monocytes.**

A-D. Ranked bar plot showing the log2 fold changes of immune cell proportions between OP and healthy controls in the four datasets. Correlations with |Pearson's correlation coefficient| ≥ 0.8 and p-value ≤ 0.01 are displayed. E. Association of cell infiltration and immune related gene expression in four datasets. Red color indicates positive correlation and blue indicate negative correlation. F. Association of cell infiltration and osteoclast differentiation related gene expression in four datasets. Red color indicates positive correlation and blue indicate negative correlation.

**Supplementary Figure 3. Construction of ceRNA network of circRNA and immune-related cytokine genes in osteoporosis.**

A. Based on the integration results of the four algorithms, the bar plot showing the number of exons contained in circRNAs. B. Histogram showing the distribution of genes with different number of detected circRNAs. C. Histogram showing the distribution of circRNAs with different length. D. The bar graph showing the number of candidates circRNA with different type. E. The bar graph showing the number of candidates circRNA located on different genomic regions. F. The heatmap diagram showing the expression profile of DE circRNAs.

**Supplementary Figure 4. Single-cell data validate the changes in immune cell proportions and the upregulation of cytokine gene expression in osteoporotic patients.**

A. Gene expression level of key markers of different cell types was represented with UMAP plot. B-C. Dot plot showing the expression patterns of osteoclast differentiation-related genes across various immune cell types and sample group.

## 2. Supplementary **Tables**

**Supplementary Tables 1. Clinical detection of mRNAs, circRNAs and NK cells [(** $\bar{\boldsymbol{x}}\boldsymbol{\pm}\boldsymbol{s}$ **) , *M*(*P_25_* , *P_75_*)]**

| Variables | HC (*n*=7) | PMOP (*n*=7) | SOP (*n*=7) | $\eta_{p}^{2}$ | *F* / *H* | *P* | HC vs PMOP | | PMOP vs SOP | |
| --- | --- | --- | --- | --- | --- | --- | --- | --- | --- | --- |
|  |  |  |  |  |  |  | *P* | 95%CI | *P* | 95%CI |
| *AIF1* | 0.77(0.75,2.34) | 3.23(2.88,3.39) | 1.53(0.9,2.61) | 0.60 | 12.85 | 0.002 | 0.000 | - | 0.014 | - |
| *CX3CR1* | 1.30±0.92 | 2.71±1.16 | 5.28±1.76 | 0.64 | 16.19 | ＜0.001 | 0.061 | (-2.91, 0.08) | 0.002 | (1.08, 4.06) |
| *FCGR3A* | 1.16±0.66 | 2.17±1.70 | 4.94±0.86 | 0.69 | 19.77 | ＜0.001 | 0.122 | (-2.31, 0.30) | ＜0.001 | (1.46, 4.07) |
| *SPON2* | 0.79(0.68.09) | 1.43(0.18,3.44) | 4.57(3.57,6.10) | 0.57 | 12.32 | ＜0.001 | 0.897 | - | 0.012 | - |
| *C3AR1* | 1.03(0.88,1.31) | 4.67(1.13,4.89) | 6.37(6.03,9.25) | 0.73 | 15.15 | ＜0.001 | 0.182 | - | ＜0.001 | - |
| *STAT1* | 1.20±0.72 | 1.75±1.26 | 4.181±0.85 | 0.68 | 18.75 | ＜0.001 | 0.300 | (-1.64, 0.54) | ＜0.001 | (1.34, 3.52) |
| *IL2RA* | 1.37±0.92 | 4.75±0.65 | 5.17±2.33 | 0.60 | 13.57 | ＜0.001 | 0.001 | (1.70,5.06) | 0.605 | (-2.10, 1.26) |
| *TBX21* | 1.34±1.01 | 6.55±1.40 | 5.45±2.63 | 0.64 | 16.03 | ＜0.001 | ＜0.001 | (3.17,7.25) | 0.272 | (-3.14, 0.94) |
| hsa-TMEM55A_0001 | 1.2(0.46,1.9) | 0.24(0.04,0.38) | 0.02(0.02,0.1) | 0.78 | 16.03 | ＜0.001 | 0.017 | - | 0.110 | - |
| hsa-TNFRSF21_0001 | 1.39±0.95 | 4.32±1.43 | 5.58±2.58 | 0.53 | 10.08 | 0.001 | 0.007 | (0.92,4.94) | 0.206 | (-3.27, 0.75) |
| hsa-KMT5B_0001 | 0.84(0.69,2.66) | 3.41(3.14,6.22) | 6.85(4.23,10.55) | 0.63 | 13.29 | 0.001 | 0.028 | - | 0.155 | - |
| NK% (of lymphocytes) | 12.45±5.86 | 9.11±6.08 | 21.12±9.42 | 0.36 | 5.04 | 0.018 | 0.403 | (-4.86, 11.55) | 0.007 | (3.81,20.21) |
